# Exploring Area-Dependent Pr_0.7_Ca_0.3_MnO_3_-Based Memristive Devices as Synapses in Spiking and Artificial Neural Networks

**DOI:** 10.3389/fnins.2021.661261

**Published:** 2021-07-02

**Authors:** Alexander Gutsche, Sebastian Siegel, Jinchao Zhang, Sebastian Hambsch, Regina Dittmann

**Affiliations:** Peter Grünberg Institut (PGI-7/10), Forschungszentrum Jülich GmbH & JARA-FIT, Jülich, Germany

**Keywords:** PCMO, memristive devices, perceptron learning, resistive switching, multilevel switching

## Abstract

Memristive devices are novel electronic devices, which resistance can be tuned by an external voltage in a non-volatile way. Due to their analog resistive switching behavior, they are considered to emulate the behavior of synapses in neuronal networks. In this work, we investigate memristive devices based on the field-driven redox process between the p-conducting Pr_0.7_Ca_0.3_MnO_3_ (PCMO) and different tunnel barriers, namely, Al_2_O_3_, Ta_2_O_5_, and WO_3_. In contrast to the more common filamentary-type switching devices, the resistance range of these area-dependent switching devices can be adapted to the requirements of the surrounding circuit. We investigate the impact of the tunnel barrier layer on the switching performance including area scaling of the current and variability. Best performance with respect to the resistance window and the variability is observed for PCMO with a native Al_2_O_3_ tunnel oxide. For all different layer stacks, we demonstrate a spike timing dependent plasticity like behavior of the investigated PCMO cells. Furthermore, we can also tune the resistance in an analog fashion by repeated switching the device with voltage pulses of the same amplitude and polarity. Both measurements resemble the plasticity of biological synapses. We investigate in detail the impact of different pulse heights and pulse lengths on the shape of the stepwise SET and RESET curves. We use these measurements as input for the simulation of training and inference in a multilayer perceptron for pattern recognition, to show the use of PCMO-based ReRAM devices as weights in artificial neural networks which are trained by gradient descent methods. Based on this, we identify certain trends for the impact of the applied voltages and pulse length on the resulting shape of the measured curves and on the learning rate and accuracy of the multilayer perceptron.

## Introduction

Most modern computer architectures are based on the von Neumann principle, which separates the data processing unit from the data storage. As the performance of processors increased strongly over the last decades, the bandwidth for the communication between processor and data storage became the limiting factor for the overall computational performance. This is called the von Neumann bottleneck (Backus, 1978) (Wolf and McKee, 1994).

The limit is especially problematic for tasks, where simple operations are performed on large sets of data, e.g., learning tasks in massively parallel systems mimicking brain-like functionalities or vector-matrix multiplications in artificial neural networks (ANNs) during the inference step. The multiplication of the input nodes of a layer with the weight matrix yields the output of this layer. A possible strategy to overcome this von Neumann bottleneck for ANNs is the usage of resistive arrays as weight matrices (Xia and Yang, 2019). To achieve tunable weights, one approach is the so-called memristive device, an electrically tunable resistor. Previous works already show that memristive crossbar arrays allow for efficient vector-matrix multiplication (Cai et al., 2019). The use in ANNs was demonstrated on many network types such as single-layer perceptrons (Alibart et al., 2013; Prezioso et al., 2015) as well as multilayer perceptrons (Moon et al., 2015; Burr et al., 2017; Babu et al., 2018; Go et al., 2019; Wu et al., 2020) and convolutional neural networks (CNNs) (Yakopcic et al., 2017). Many groups show that memristive devices can already today replace conventional networks trained in software for many applications. Li et al. report a recognition accuracy of more than 97% on the MNIST dataset, which is common for benchmarking of pattern recognition tasks. Also, more complex tasks like face recognition have been demonstrated (Yao et al., 2020). These similar network performances are often achieved at higher-energy efficiencies and make memristive device-based ANNs most useful for low-energy applications at the edge and in the IoT sector (Chowdhury et al., 2018) (Krestinskaya et al., 2020). A large variety of different types of memristive devices have been proposed for neuronal networks so far in the literature mimicking behavior of biological synapses like, e.g., long-term potentiation and depression (LTP/LTD) and even more complex aspects of synaptic plasticity like simple forms of spike timing dependent plasticity (STDP), but no optimal memristive device type has been identified yet. For a given choice of materials, the ANN, the learning rule and the update rule have to be adjusted to obtain best performance. In this work, we propose an update rule for a specific memristive device based on Pr_0.7_Ca_0.3_MnO_3_ (PCMO) after a thorough investigation of its switching behavior and the influence of different material stacks.

In memristive devices, information is stored by the change in the resistance that can be switched by an applied bias in a non-volatile manner. Different mechanisms and materials that show resistive switching have been reported in literature (Simmons and Verderber, 1967; Asamitsu et al., 1997; Sawa, 2006, 2008; Tsymbal and Kohlsted, 2006; Jooss et al., 2007; Waser et al., 2009; Herpers, 2014). In this work, we will address the mixed valence manganite (PCMO) in combination with a tunnel oxide that has been either deposited directly by physical vapor deposition or that has been formed by the redox process with an oxidisable metal top electrode. Combinations of PCMO with many different metals are reported in literature so far: e.g., Al (Seong et al., 2009a), Ta (Seong et al., 2009b), Ti (Seong et al., 2009b), W (Liu et al., 2011), and others (Moon et al., 2014, 2015; Baek et al., 2017; Go et al., 2019). It is proposed that the field-driven movement of oxygen anions between the PCMO layer and the reactive metal electrode is the underlying switching mechanism (Sawa et al., 2004; Asanuma et al., 2009; Seong et al., 2009a).

PCMO is known for its area-type resistive switching properties, namely that the change of the resistance happens over the whole device area (Herpers, 2014; Bagdzevicius et al., 2017). Since the current of the area-type switching devices scales for both the high resistive state (HRS) and the low resistive state (LRS) with the device area, the resistance values can be adapted to the given circuit requirements. This is not the case for the most common filamentary-type memristive devices. Moreover, filamentary-type switching is usually indicated by a sharp SET process. In contrast, area-type switching devices exhibit a gradual SET and RESET that enhances their ability for analog switching in comparison with filamentary memristive devices. Due to their analog switching behavior, PCMO-based resistive switching devices are considered hardware representation for synapses in artificial neural networks as described above. In particular, it has been shown that they can emulate aspects of synaptic plasticity (Park et al., 2012, 2013, 2015; Moon et al., 2014; Fumarola et al., 2018).

In this work, we compare in detail the performance and analog behavior of PCMO-based devices with different interface configurations. In particular, we compare the more common Al/PCMO devices with a natively formed Al_2_O_3_ oxide to devices with a directly sputtered Ta_2_O_5_ and WO_3_ as interface layer. For all devices, we can demonstrate analog switching behavior. We demonstrate a STDP-like behavior on single PCMO devices. This learning rule for spiking neural networks (SNN) stems from neuroscience and neurophysiology. Furthermore, we investigated in detail the impact of the material stack as well as pulse length and height on the shape of the analog stepwise SET and RESET curves. This stepwise change of conductance mimics aspects of LTP/LTD of biological synapses. We use the experimental data as input for simulations of the training of a multilayer perceptron for pattern recognition and reveal how the different electrical stimuli and the resulting shapes of the stepwise SET and RESET measurement (SPM and RPM) curves affect the learning rate and the accuracy of the network based on a gradient descent learning rule, which is a learning rule for conventional ANNs. Comparing STDP and gradient decent methods, STDP only requires local information processing between the two neurons adjacent to the very synapse, while gradient descent methods take the global error of the complete network into account. Here, we present how both learning rules can be achieved with the same memristive device.

## Experimental

### Sample Preparation

**FIGURE 1:**
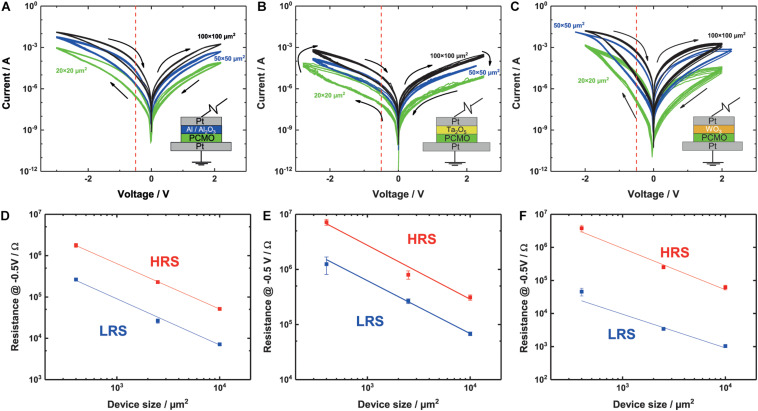
**(A–C)** I-V curves for three different materials, **(B)** Al/Al_2_O_3_, **(C)** Ta_2_O_5_, and **(D)** WO_3_, like it is indicated by the layer stack in the lower right corner. The voltage is applied on the top electrode. For every device, three different sizes—100 × 100, 50 × 50, and 20 × 20 μm^2^—are measured. The red dotted line indicates the read out voltage for the area scaling. The switching voltages differ between the three stacks: **(A)** Al_2_O_3_: 2.2 V/–3 V, **(B)** Ta_2_O_5_: ± 2.5 V, and **(C)** WO_3_: ± 2 V. **(D–F)** Area dependence of the LRS and HRS. The resistance value is scaling with the device size, for all of the three materials, **(D)** Al_2_O_3_, **(E)** Ta_2_O_5_, and **(F)** WO_3_. The slopes of the linear fit for all of the devices and the HRS and LRS are around –1 Ω/μm^2^. Slopes can be seen in Table 2.

The memristive devices consist of a 25-nm-thick Pt bottom electrode, a 20-nm PCMO film grown by pulsed laser deposition (PLD), a 7-nm-thick interface layer, either Al, Ta_2_O_5_ or WO_3_ and a 25-nm-thick Pt top electrode as sketched in the insets of Figures 1A–C. The Pt layer that serves as bottom electrode is DC sputtered on top of a 5 nm Ta adhesion layer on a thermally oxidized Si wafer.

The PLD growth of PCMO is performed with an O_2_ pressure of 0.133 mbar at room temperature (RT). A laser fluence of 1.33 J/cm^2^ and a frequency of 5 Hz are used during PLD growth. Around 2,800 pulses are needed to grow a 20-nm amorphous PCMO layer. Afterward, the PCMO thin film is annealed in N_2_ atmosphere at 650°C for 2 min in order to crystallize the PCMO layer.

The Ta_2_O_5_ and WO_3_ layers are deposited by RF sputtering at RT. Both depositions are performed at 200 W with 5 × 10^–2^ mbar pressure and an Ar/O_2_ ratio of 3/2. Afterward, the sample is transferred *in situ* into an e-beam evaporator to deposit the Pt top layer which is used as top electrode. During the Pt deposition in vacuum, the e-beam process heats the sample up to 180°C. For the Al device stack, a 7-nm layer is also deposited on top of the PCMO layer by e-beam evaporation and capped *in situ* with the Pt layer. During the short deposition of the Al layer, no significant increase in temperature can be detected. Here also, a 25-nm Pt capping layer is used.

The patterning of the top electrode and the active interface layer of the devices is performed by optical lithography and Ar ion-beam etching. The pad size varies between 100 × 100, 50 × 50, 20 × 20, and 10 × 10 μm^2^.

### Electrical Measurements

In preparation of the electrical measurements, the samples are glued to a large sample carrier chip with Pt pads. The BE is contacted to one of the Pt pads on the sample carrier using aluminum wire bonding. Two different setups are used to characterize the samples electrically, namely one to perform the quasi-static current-voltage (I-V) measurements, the other one to apply pulses to the devices. A Keithley 2611B is used to measure the I-V characteristics of the devices. The connection between the measurement unit and the device is performed by soft tungsten needles. Every measurement starts with an initialization curve: 0 V→2.5 V→–2.5 V→0 V. During this initialization procedure, the oxide layers of the metal are presumably homogenized (Arndt et al., 2017). Afterward, the regular switching cycle can be performed: 0 V→RESET voltage (positive)→SET voltage (negative)→0 V. The SET and RESET voltages have to be adapted for the different interface layer materials. In Table 1, the writing voltages that show the most stable switching for the different interface layers can be found along with the read voltage.

**TABLE 1:** Voltages for the conducted measurements.

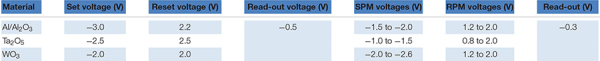

**TABLE 2:** Slope of the linear fit of the resistance vs. area plot for all the different materials.

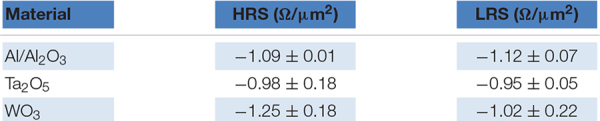

The pulse measurements for the multilevel SPMs and RPMs are performed with a Keithley 4200A. Different pulse lengths between 1 and 100 μs are employed. A large variety of combinations of SPM and RPM voltages are investigated. The parameter ranges that are used for the different devices are displayed in Table 1.

The STDP measurements are performed with an Arc One from Arc Instruments. For all three device types, a pulse length of 100 μs with a pulse voltage of 2 V/–2 V is investigated.

## Electrical Characterisation of PCMO Memristive Devices

### Quasi Static I-V Measurements

In Figure 1A, the I-V measurements of a typical sample with an Al interface layer can be seen. A clear hysteresis of the I-V curve on both the positive and the negative branch is visible. The SET takes place at negative voltages and the RESET at positive voltages. For negative applied voltages, the difference between the LRS and the HRS, called ON/OFF-ratio is higher. Concerning the gradual switching of the area type switching devices, no distinct SET or RESET voltage can be defined. Therefore, we always choose a voltage pair that allows stable switching of the devices without any change of the I-V curves during the repeated switching, e.g., 2.2 and 3 V for RESET and SET, respectively, in case of the Al devices. For simplicity reasons we will call the maximum voltage in the different voltage directions SET and RESET. During the RESET, the slope changes at 1.8 V. A similar but smaller change in slope can be seen during the RESET at −2 V. Furthermore, the RESET and SET are both gradual, with no abrupt jumps into the HRS or LRS. The I-V curves for different pad sizes all have the same shape with smaller differences, like the opening on the positive side. For smaller devices, the opening becomes smaller in the positive branch. This effect is not observed for the negative branch.

The I-V curves of a device with a Ta_2_O_5_ interface layer are shown in Figure 1B. This switching polarity is the same as for the Al devices, and HRS and LRS are clearly separable on the negative side. Also, a change in the slope of the I-V curve can be found around –2 V. On the positive branch, no opening and no change in slope can be seen.

The WO_3_ devices show a different shape of the I-V curve compared with the Al and Ta_2_O_5_ devices. The positive and the negative branches both show two clearly separable resistive states, see Figure 1C. In contrast to the case of Al and Ta_2_O_5_ devices, the I-V curves are very symmetric for positive and negative polarities. In particular, the increase in current in the LRS state with voltage in the negative branch is much higher, compared with Figures 1A,B. For the WO_3_ devices, a stable switching curve can be found with symmetric switching voltages at ± 2 V. At around –1.8 V, a change in the slope can be seen at least for the 50 μm × 50 μm and the 100 μm × 100 μm devices. For the 20 μm × 20 μm devices a similar change in slope can be surmised, but not clearly determined.

Each device state, HRS and LRS, for the Al, Ta_2_O_5_, and WO_3_ interface devices are tested regarding their retention time. Over a period of several days, no change in the states can be determined. The samples are stored at room temperature and in ambient atmosphere.

To prove that all of the devices show area type resistive switching, we read out the resistance at –0.5 V since switching effects can be excluded at this voltage and the resistance at this voltage is plotted against the device area (see Figures 1D–F). The read out is chosen to be on the negative branch due to a higher ON/OFF ratio. A clear linear relationship between the device resistance and the device area can be seen for the HRS and the LRS for all of the devices with a slope around −1 Ω/μm^2^, as expected by Ohm’s law. The exact values of the fitted slopes can be found in Table 2.

**FIGURE 2:**
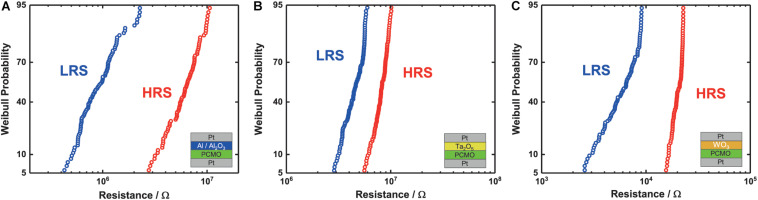
**(A**–**C)** The Weibull plot of the combined cycle to cycle variability (c2c) and device-to-device variability (d2d) for the three different stacks, **(A)** Al_2_O_3_, **(B)** Ta_2_O_5_, and **(C)** WO_3_, indicated by the stack in the lower right corner. For each stack, 10 different devices with each up to 100 cycles have been investigated.

Additionally, we studied the device-to-device (d2d) and cycle-to-cycle (c2c) variability of the devices during the quasi-static I-V measurement. For these measurements, we used the 20 μm × 20 μm devices. Figures 2A–C shows the combined c2c and d2d Weibull distribution for the different devices, namely, (A) Al/Al_2_O_3_, (B) Ta_2_O_5_ and (C) WO_3_. For the Al/Al_2_O_3_ interface layer, it can be seen that the HRS and LRS are clearly separable over their whole resistance range. The spread of the HRS and the LRS is half an order of magnitude. For the Ta_2_O_5_ interface layer (Figure 2B), the spread for the different devices and cycles is smaller. However, due to the smaller ON/OFF ratio, the overlap of the two states is around a few percent. For the WO_3_ devices, the variability plot (see Figure 2C) differs from the plot of the other devices. It can be seen that the LRS shows a much higher variability than the HRS. The variability of the HRS is as small as for all of the other device types. Comparing all three device stacks, the Al/Al_2_O_3_ devices show a higher ON/OFF ratio than the Ta_2_O_5_ devices and a lower variability than the WO_3_ devices.

### Spike Timing Dependent Plasticity

In STDP, the change of a synaptic weight between neurons depends on the time difference between two spikes, the pre- and post-synaptic neuron pulse. The memristive devices act as synapses, and the pre- and post-synaptic pulse are applied at the top/bottom electrode, respectively.

**FIGURE 3:**
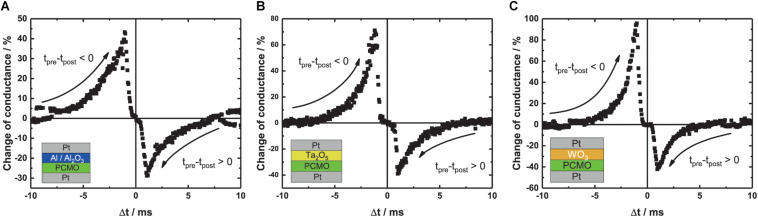
Relative change of conductance for different time delays between the pre- and post-synaptic pulse during STDP measurement for the three different devices. **(A)** Al/Al_2_O_3_, **(B)** Ta_2_O_5_, and **(C)** WO_3_.

Figure 3 shows the relative change in conductance of the three different memristive devices for different time delays between the pre- and post-synaptic pulse. All devices show an increase/decrease in conductance for a negative/positive time delay between the pulses, respectively. The Al STDP curve (Figure 3A) shows a symmetric increase or decrease of the conductance for the time delay between the pulses compared with the STDP curves of the Ta_2_O_5_ and the WO_3_ (Figures 3B,C). The WO_3_ (Figure 3C) shows a clear asymmetry between the increase and decrease of conductance. The maximum increase in conductance is around twice as high as the decrease. Therefore, all three types of devices are suitable for the implementation in SNNs based on the STDP learning rule.

### Stepwise SET and RESET Pulse Measurements

We perform stepwise SET and RESET pulse measurements by applying the same voltage pulse multiple times to one device without switching the device back into a predefined state. By applying pulses with a lower voltage, compared with the voltages used during the IV measurement, it is possible to tune the resistance of the devices in a gradual way between the HRS and the LRS and vice versa. The transition from the HRS to the LRS in the SPM and the transition from the LRS to the HRS in the RPM happen stepwise. With these measurements, we can show that it is possible to write different resistance states into the investigated devices, resembling the LTP/LTD behavior of biological synapses.

**FIGURE 4:**
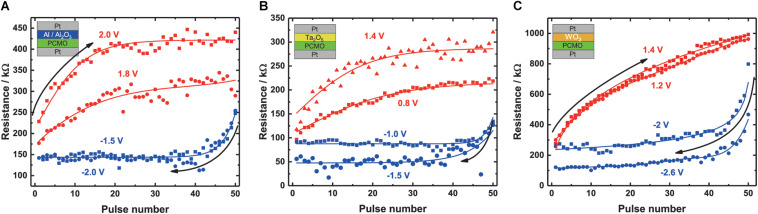
Results of the SPM and RPM for the different devices. The RPM curve (red) and the SPM curve are plotted for the same material into the same coordinate system. The SPM curve starts at higher pulse numbers and goes down to lower pulse numbers; the RPM curve can be read as normal. The pulse voltages for the different stacks can also be seen in Table 1. **(A)** Al_2_O_3_, **(B)** Ta_2_O_5_, and **(C)** WO_3_. Every pulse has a length of 100 μs. For the shown curves, the fits are shown in the plots.

In Figure 4A, the SPM and RPM measurements of PCMO with the Al/Al_2_O_3_ interlayer are depicted. The chosen voltage for the SPM and RPM are 1.8 V/2.0 V and –1.5 V/2.0V, respectively, at a pulse length of 100 μs. Every pulse was applied 50 times without going back to the initial state. The largest resistance change for the SPMs and RPMs of the Al/Al_2_O_3_ interface device occurs during the first few pulses of a cycle. For the positive voltage curves, the resistance saturates after ∼20 pulses for both voltages but with different saturation level, a higher/lower resistance for the higher/smaller voltage, respectively. Furthermore, the increase in resistance at the beginning of the curve is higher with a higher pulse voltage and smaller with smaller pulse voltage. After the steep increase in the beginning, the resistance only slightly increases. The SPM curves that are measured with positive pulse voltages saturate after ∼10 pulses. Both curves show a clear non-linear behavior.

The SPM and RPM curves of the Ta_2_O_5_ device are depicted in Figure 4B. The RPM curve is shown for two different pulse voltages, namely, 0.8 and 1.4 V, each with 100 μs pulse length. The SPM pulses have a height of –1.0 or –1.5 V, also with a pulse length of 100 μs. Again, both the RPM and SPM curve characteristics are non-linear. The SPM curves saturate after ∼10 pulses, similar to the Al/Al_2_O_3_, but the maximum resistance reached is different. The larger negative voltage leads to a lower resistance value, compared with the smaller negative voltages. For the RPM curves, the resistance increases less with each pulse for the 0.8 V pulses as for the 1.4 V pulses. Furthermore, the obtained saturation resistance is also smaller and therefore the ON/OFF ratio is smaller. Beside the smaller ON/OFF ratio, the curve shows a more linear increase in resistance during the pulse measurement with the smaller SET voltage compared with the larger SET voltage.

In Figure 4C, measurements for the WO_3_ stack with 1.2 V/1.4 V and –2 V/–2.6 V for RPM and SPM, respectively, are shown. All measurements are performed with a pulse length of 100 μs. The RPM curves of WO_3_ are more linear compared with the RPM curves of the Al and the Ta_2_O_5_ devices, and no clear saturation can be seen for the shown RPM curves. The SPM curves show a saturation after ∼20 pulses with a slight resistance decrease afterward. Here, a clear separation between the saturation levels for the different voltages can also be seen.

### Behavioral Modeling of the Resistance Changes of the PCMO Devices

To better analyze the impact of the material stack and applied voltages on the shape of the SPM and RPM pulse measurements and to use these measurements in the ANN simulations in Section “Perceptron Learning of Mnist Dataset,” the evolution of the resistance for the devices with an Al, Ta_2_O_5_, and WO_3_ interlayer is mathematically fitted. Similar to other approaches in literature (Suri et al., 2015), a logistic function

(1) $$y\,\left(n\right)=\frac{\overset{´}{\text{y}}}{1+\text{exp}\,\left(-\alpha \times n-c\right)}$$

is employed, where *y* is the fitted resistance for the RPM curve and conductance for the SPM curve. ý is the maximum value at which the function saturates, and α and *c* determine the steepness of the increasing swing. In the following, parameters concerning the SPM fit are equipped with the index *SPM* and parameters concerning the RPM fit with the index *RPM*. This formula shows a strong saturation for high values of the pulse number *n* as observed in our experiments and reasonably good fitting of the measured resistance values. A fitted resistance value can therefore be attributed to each measured resistance, leading to a total of 50 different resistance values for every SPM and RPM curves and therefore in total 100 resistance levels for every material stack and pair of SPM and RPM voltages. The different resistance levels are not evenly spaced.

The fit function is used to determine the change of the resistance of a memristive device upon the application of either a SET or RESET pulse. For a SET pulse, the fit function for the respective SPM curve *y*_SPM_(*n*) is inverted and the current conductance (before the update pulse) of the device is used to determine the pulse number *n*_current_, which resembles this conductance. Following to this, *y*_SPM_(*n*) is evaluated at *n*_current_ + 1 to yield the conductance of the device after the SET update pulse. For a RESET pulse, the inverse of the RPM curve’s fit function *y*_RPM_(*n*) and the current resistance yield *n*_current_ and *y*_RPM_(*n*_current_ + 1) gives the resistance value of the device after the update pulse.

Another common approach for a behavioral model in the literature is fitting the resistance change [e.g., (Suri et al., 2015)] instead of the actual resistance as it is proposed in this work. However, this approach showed a similar fitting accuracy for the data used here but a lower computational performance in the TensorFlow environment.

The fit parameters ý_SPM_ and ý_RPM_ correspond to the saturation value of the resistance in the SPM and RPM, respectively. Therefore, the maximum ON/OFF ratio of a pair of SPM and RPM curves can be calculated from these parameters. The ON/OFF ratio strongly depends on the used material stack. With the Ta_2_O_5_ interlayer samples, the lowest ON/OFF ratios can be reached, while the WO_3_ samples show the highest values and with an Al interlayer, intermediate values can be reached. Additionally, higher SET and RESET voltages lead to a higher maximum ON/OFF ratio in the pulse measurements, except for the SPM with –2.6 V for the WO_3_ interlayer samples and the 1.6-V RPM for the Ta_2_O_5_ interlayer samples. It can be found that an increase of the SET voltage leads to a lower saturation resistance in the SPM and a higher RESET voltage to a higher saturation resistance of the RPM.

For the α parameter of the fit function (1), which resembles the steepness of the initial change of the resistance, no clear dependency on the applied voltage can be found, neither for the SPM nor the RPM. Concerning the α_SPM_ parameter for the fit of the SPM, a clear separation between the different material stacks can be observed, where the Al interlayer samples show the highest, Ta_2_O_5_ intermediate and WO_3_ the lowest values. In the fit of the RPM curves, a trend to increasing α_RPM_ parameters with an increasing reset pulse voltage can be observed. However, the height of the increase is the largest for the Ta_2_O_5_ interlayer samples and comparably small for the WO_3_ interlayer samples.

In conclusion for the fit parameters, in the most cases, a higher SET voltage leads to a lower saturation resistance in the SPM and a higher RESET voltage to a higher saturation resistance in the RPM. α_SPM_, corresponding with the steepness of the initial increase of the SPM, depends mostly on the material and not on the SET voltage, while α_RPM_, for the RPM steepness, depends on the RESET voltage.

### Impact of Pulse Length on SET and RESET Pulse Measurements

**FIGURE 5:**
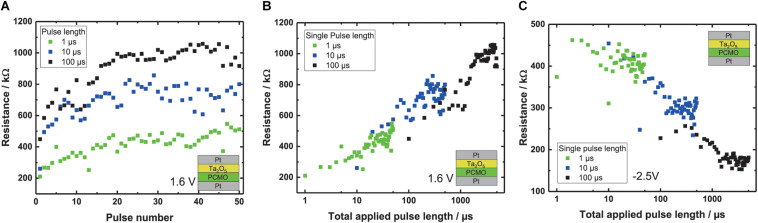
**(A)** RPM of the Ta_2_O_5_ devices with 1.6 V per pulse and different pulse lengths –1, 10 and 100 μs. **(B)** Resistance of the RPM plotted against the total applied pulse length for three different pulse lengths with a pulse voltage of 1.6 V. **(C)** SPM curves of the Ta_2_O_5_ devices with the resistance plotted against the total applied pulse length, with a pulse voltage of –2.5 V.

In order to study the impact of the pulse length on the shape of the SPM and RPM curves, we vary the pulse length between 1 μs and 100 μs. Figure 5A shows that using shorter pulses for the RPM, the maximum reached resistance after 50 pulses is smaller, similar to what we have observed for smaller pulse amplitudes (see Figure 4B). Moreover, Figure 5A implies that the total change of the resistance only depends on the total time of the applied pulses irrespective of the length of a single pulse. For example, for the application of a single pulse of 100 μs or 10 pulses of 10 μs pulse length, the total applied time is the same and the observed resistance change is the same. Figure 5B shows the measured device resistance at –0.3 V, plotted against the total applied pulse length for the Ta_2_O_5_ RPM curve starting in the LRS. A clear trend of increasing resistance with increasing total applied pulse time can be seen. Also the measurements with the different pulse lengths show a continuous behavior. For the Ta_2_O_5_ devices, this behavior is also depicted for the SPM curve in Figure 4C. This proves that the total change in resistance is indeed only dependent on the total applied time for Ta_2_O_5_. This behavior is also observed for the Al and the WO_3_ devices (not shown here).

In summary, the combination of different pulse lengths and pulse voltages makes the devices flexible in the resistance range that can be used and the resistance change that one pulse triggers. In addition to the former described current adaptability of the devices by their sizes, this gives the opportunity to adapt the pulse length to the network requirements.

### Discussion of the Impact of Pulse Height and Length on SPM/RPM

As described in the previous section, the final ON/OFF ratio that can be reached after saturation increases with the pulse height. The tradeoff is to find a voltage that allows a high saturation ON/OFF ratio but without reaching the saturation immediately, which would lead to a binary synapse. With respect to the pulse length dependence (see Figure 5A), the reduction of the pulse length results in a smaller increase in resistance and the final ON/OFF ratio that can be reached after saturation increases only with the pulse height and not with the pulse length. To avoid switching the device completely with only one pulse, the voltage and the applied pulse time has to be reduced. This way we can reach a high number of intermediate resistance states.

In filamentary systems, the switching current is confined to the filament resulting in high current densities and self-heating up to 800 K (Menzel et al., 2011). The increase in temperature leads to a to a self-enhanced, abrupt SET process. In area type switching, the current is distributed over the whole area resulting in low current densities and a large dissipation area. Simulations confirm that self-heating is not important in area type devices, and we neglect its influence on the switching process (Menzel et al., 2019). As a result, the velocity of the oxygen ions only depends on the force of the applied electric field and the diffusion force. It does not depend on the length of the applied voltage pulse.

We assume that the resistance changes with the amount of oxygen in the tunnel oxide and in the PCMO. An oxygen ion transfers from the tunnel oxide into the PCMO (or vice versa) if it overcomes the distance to the interface between the two materials. If we apply an electric field, oxygen ions begin to move. The higher the distance each oxygen ion can travel, the more ions can move in total from the tunnel barrier to the PCMO (or vice versa) if the drift process takes place *via* vacancy sites. Therefore, the change in resistance is directly related to the total distance an oxygen ion has moved.

This distance is the time integral of the velocity that only depends on the total time a voltage is applied as long as the velocity itself is not a function of the pulse length. This should be the case if no Joule heating takes place. Therefore, the distance the oxygen can be moved does not depend on the number of pulses and their length. For example, one 100 μs pulse has the same effect as ten 10 μs pulses (see Figures 4B,C).

## Perceptron Learning of Mnist Dataset

The presented devices allow for usage with two different learning rules. The measurements presented in Section “Spike Timing Dependent Plasticity” resemble a synaptic STDP behavior and therefore suggest the use of the proposed devices for Hebbian style learning in a spiking neural network. The second approach uses the stepwise resistance change in the pulse measurements presented in Section “Stepwise SET and RESET Pulse Measurements”. The gradual nature of the resistance change can be exploited in an artificial neural network, which is trained by a gradient descent learning rule. This duality shows the wide range of applications for the proposed devices in neuromorphic systems, as these learning rules differ significantly in the scope of processed information (local comparison of pre- and post-synaptic activity for Hebbian learning and global error minimisation for gradient descent) and the initial point of their derivation (neurophysiology for Hebbian learning and mathematical optimisation theory for gradient descent). In the following, an exemplary ANN trained by a gradient descent learning rule is shown.

To investigate the use of PCMO resistive switching devices as presented above as weights in an ANN, we conduct simulations of multilayer perceptrons in a TensorFlow (Abadi et al., 2016) environment in Python. Furthermore, the impact of the different material stacks and the hyperparameters SET/RESET pulse voltage and pulse length on the learning and recognition accuracy are analyzed. To compare and benchmark the results of our network, the common dataset of hand-written digits MNIST is used for training of the network and validation of the recognition performance.

### Gradient-Descent Learning of the MNIST Dataset

The investigated perceptron network consists of four layers of neurons with the second and third being hidden. The input layer has 784 neurons, the first hidden layer 250, the second hidden layer 125 and the output layer 10 neurons. This structure is chosen to make the network comparable with similar memristive networks in the literature. Similar to previous works, a matrix structure of PCMO-based ReRAM devices is assumed as the weight layer between two neuron layers (Xia and Yang, 2019). A more detailed description of the network can be found in the Supplementary Material. The weights are initialized randomly from the range the employed SPM and RPM curves provide. In order to determine whether a SET or RESET pulse must be applied to a ReRAM device, a form of gradient descent learning using the backpropagation algorithm is employed.

In the forward pass, a sample image is presented to the input of the network with the grayscale values of each pixel being converted to an input voltage. By Ohm’s and Kirchhoff’s Law, this vector of input voltages is transformed to a vector of output currents by the memristive weight matrix. A Rectified Linear Unit (ReLU) function determines the input voltage to the next weight layer from these currents.

The gradient for the gradient-descent algorithm is calculated in the backward pass of the total network error E for every weight as $\frac{\partial E}{\partial g_{i,j}}$. This calculation is executed within the TensorFlow framework. The calculated gradients for every weight are accumulated within each epoch. In every training epoch, a subset of 60,000 samples from the MNIST dataset is shown to the network, resulting in a batch size of 60,000. Previous works by Gao et al. (2020) showed that larger batches can lead to a better recognition performance. With this large batch size, only one update cycle per epoch is performed.

In the update operation of the PCMO devices we propose here, either a gradual SET, RESET, or no pulse can be applied to a device. The pulse height for SPM and RPM are fixed. Therefore, only the sign of the accumulated gradient would determine whether a device receives a SET or RESET pulse for update. With this sign update rule, an infinitesimal small gradient would have the same effect as a large gradient, what can be expected problematic for the training of the network. Therefore, the set of updated conductances is restricted to only the largest positive gradient and the smallest negative gradient in every layer. Another benefit of such a very sparse update matrix in a matrix-shaped weight layer is that it is much more time consuming to update a large number of conductances than to infer the whole network. Using a matrix structure of the PCMO devices, the inference of a complete layer takes one step, whereas the update of device is performed sequentially. The very low number of updated devices on the other hand leads to a large number of learning epochs necessary to reach the maximum recognition accuracy.

After each learning epoch, a validation subset of 10,000 samples from the MNIST dataset, which is different than the learning set is shown to the network, and the fraction of correctly recognized numbers is calculated as the recognition accuracy after this epoch. To investigate the impact of the PCMO-based ReRAM devices in this network, we also performed a benchmark test with the described network structure and learning rule, but floating point weights instead of resistive switching weights. In this benchmark, the network showed a maximum recognition accuracy of 96.3%.

### Results of Multilayer Perceptron Simulations With PCMO-Based Memristive Switching Weights

**FIGURE 6:**
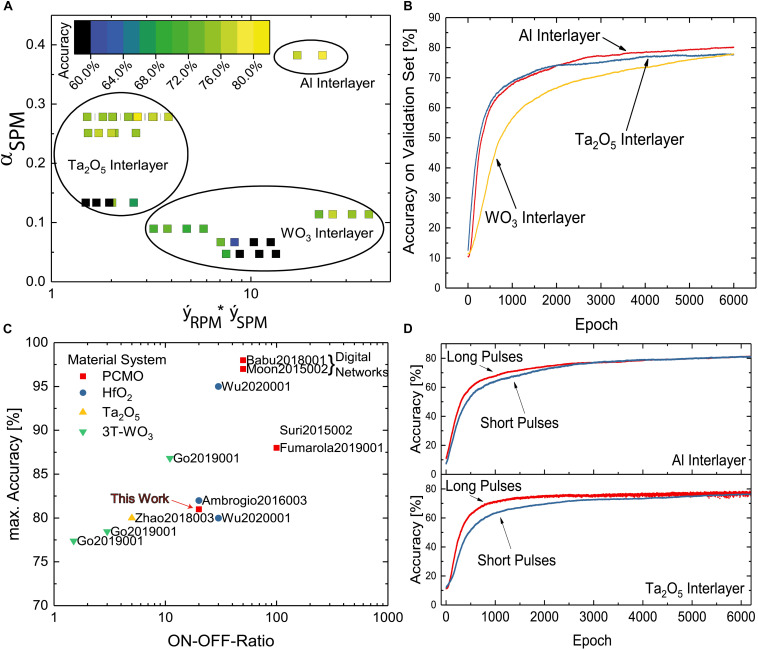
Results of the neural network simulations. **(A)** Recognition accuracy of the simulated network depending on the fitted ON/OFF ratio and the steepness of the SPM fit function. **(B)** Network recognition accuracy on a subset of the MNIST dataset. Red: Al interlayer. Blue: Ta_2_O_5_ interlayer. Yellow: WO_3_ interlayer. **(C)** Recognition accuracies of similar networks with ReRAM devices from literature depending on the ON-OFF ratio of the used ReRAM devices (Moon et al., 2015; Suri et al., 2015; Ambrogio et al., 2016; Babu et al., 2018; Fumarola et al., 2018; Go et al., 2019; Wu et al., 2020; Yin et al., 2020). **(D)** Learning curves for PCMO devices with Al and Ta_2_O_5_ interlayers with 100μ*s* (red) and 1μ*s* update pulses.

For all combinations of measured SPMs and RPMs, multilayer perceptron simulations are conducted for 6,000 epochs. As described in Section “Stepwise SET and RESET Pulse Measurements,” the material stack of the PCMO devices and choice of the voltage for the SET and RESET pulses in the pulse measurements lead to significantly different evolutions of the resistance in these measurements. This also has an influence on the maximum accuracies that can be achieved using these resistance curves for ANNs. The maximum accuracies of all simulations are plotted in Figure 6A. The *x*-axis shows the fitted maximum resistance in the SPM and RPM curves. The higher this value, the higher also the measured ON/OFF ratio in the pulse measurements is. As discussed in Section “Spike Timing Dependent Plasticity,” in most cases, a higher ON/OFF ratio can be reached by choosing higher voltages for the SPM and RPM. On the y-axis, the steepness value from the fit of the SPM α_SPM_ is shown. This parameter is mostly dependent on the used material stack (see Section “Stepwise SET and RESET Pulse Measurements”).

The lowest accuracies resulting from the training simulations can be found in the lower left corner for low ON/OFF ratio and low steepness of the SPM curve. Many of these simulations yield accuracies below 70%, and for the data points in black no learning at all with accuracies around 10% is even reached, which would be like a random drawing. However, for higher values of the α_SPM_ parameter around 0.25, the maximum accuracy increases to 75% to 80%, even for comparably low ON/OFF ratios. This means that an initially steeper increase of the conductivity of a weight leads to an increase in recognition accuracy. This observation appears contrary to previous observations that a more linear update behavior of the resistance is beneficial for the recognition accuracy (Cai et al., 2020). Burr et al. (2017) attribute this to the non-reversibility of a weight update that follows from a strong non-linearity. However, the difference here can be explained by the different update rules. With the large batch size and sparse update matrix used in this work, updates on the same device mostly happen in the same direction and update pulses with different polarities on the same device rarely occur. Therefore, the non-reversibility of a weight update is not an issue.

Another path to higher accuracies is a higher ON/OFF ratio. For a low α_SPM_of around 0.1, the accuracy increases from low to higher ON/OFF ratios. The same trend can also be found for high values of the RPM steepness factor α_SPM_ of about 0.4. The ON/OFF ratio has two effects here. Since all pulse measurements consist of an equal number of pulses, a higher ON/OFF ratio means that the difference between the resistance steps is larger, as long as the curve reaches the saturation within the same number of pulses. On the other hand, a low ON/OFF ratio of, e.g., 3 means that three OFF switched devices contribute the same activation to a neuron in the subsequent layer as one ON switched device. For higher ON/OFF ratios, e.g., of 10, 10 devices can be in the OFF state with one ON switched device still having a relatively high impact on the activation of the next neuron layer. The ability of a device to differentiate the activation of the next neuron layer decreases with decreasing ON/OFF ratio.

As described in Section 2.4, α_SPM_ parameter strongly depends on the material stack. Therefore, a separation between the materials can also be observed in Figure 6A, with the WO_3_ interlayer samples for low, the Ta_2_O_5_ interlayer samples for intermediate and the Al interlayer samples for high values of α_pot_. For each material, the complete learning curves for those pulse measurement voltages resulting in the highest maximum recognition accuracy are displayed in Figure 6B. After 6,000 training epochs, the network using the PCMO devices with the Al interlayer reaches the highest recognition accuracy with 82%. The network using the Ta_2_O_5_ interlayer devices, which initially shows a faster increase of the recognition accuracy, exhibits a saturation at a lower level of 76%. The WO_3_ interlayer devices lead to a much lower learning speed but a similar recognition accuracy of about 76%.

To benchmark the performance of the proposed network and memristive devices, the maximum accuracy reached can be compared with similar networks. In Figure 6C, a comparison of previous works on ANNs with memristive devices training MNIST is provided separated by the maximum ON-OFF ratio of the used devices on the *x*-axis. Most networks here have a similar layer and neuron-per-layer count as the network proposed in this work. The maximum accuracy of 82% reached in our simulations can be found in the lower left corner of Figure 6C, which means a 14% higher error rate compared with the same network with ideal floating point weights, as described above. It can be seen that this value is comparable with other networks using memristive devices with a similar ON-OFF ratio, which is still lower than accuracy values one would expect from conventional ANNs in software. To achieve higher recognition accuracies, the ON/OFF ratio must be increased further. With an ON/OFF ratio of around 30, Wu et al. (2020) showed an accuracy of about 95%. Even higher accuracies can be reached using memristive devices as storage for weights in a fashion of digital numbers instead of analog weights (Moon et al., 2015) (Babu et al., 2018).

As described in Section “Stepwise SET and RESET Pulse Measurements,” by varying the length of the applied pulses in the SPM and RPM, short pulses lead to slower and longer pulses to a faster progression on the SPM or RPM curve, respectively. Figure 6D shows a comparison of the learning of the MNIST dataset with long pulses of 100μs (red) and short pulses of 1μs (blue) using PCMO devices with Al and Ta_2_O_5_ interlayers. Initially, for both interlayers, the accuracy for the network using the long pulses increases faster. In the end, both saturate at about the same values, 82% for the Al interlayer devices and 78% for the Ta_2_O_5_ interlayer devices. In the simulation with the latter devices, for the long pulses, the recognition accuracy oscillates at high pulse numbers. This is not the case for the short pulses. In conclusion, the use of shorter update pulses can lead to a more stable, but slower learning process. A gain in accuracy is not reached. However, in conventional perceptron networks, the learning rate is an important factor for a successful learning and has a large impact on the convergence and accuracy of the network. In this work, we present one approach to implement a variable learning rate for resistive switching ReRAM devices by changing the pulse length. Such adaptive learning rates are not only beneficial for artificial neural networks like perceptrons but can also be used in brain-like learning systems to realize more biologically plausible learning rules from neuroscience.

In conclusion for the neural network simulations, PCMO ReRAM devices with Al, Ta_2_O_5_ and WO_3_ interlayers can be used as weights in ANN learning to MNIST dataset. For devices with an Al interlayer, the highest recognition accuracy of about 82% could be achieved. A parameter optimisation showed how the shape of the resistance evolution curve of pulse measurements affects the maximum accuracy. A high steepness of the SPM and the maximum ON/OFF ratio were identified as most important to reach the highest accuracy values. While the steepness of the SPM depends mostly on the material stack used, the ON/OFF ratio can be maximized by choosing greater voltages for the SPM and RPM. Finally, the concept of a variable learning rate was implemented using different pulse lengths and the effect on the learning speed and accuracy investigated.

## Conclusion

In this work, we compared the performance of area-dependent memristive PCMO devices with Al interlayer, where Al_2_O_3_ is formed naturally at the interface with devices where Ta_2_O_3_ and WO_3_ have been deposited intentionally. All investigated devices show area-dependent switching and exhibit a STDP-like behavior.

Furthermore, for all three types of devices, we performed SPMs and RPMs. The shape of the SPM and RPM curves differs significantly for the different materials. In particular, the WO_3_ stack showed a better linearity than the other two types of devices. Moreover, we showed that we can adapt the SPM and RPM curves with the pulse parameters. By reducing the pulse height and the pulse length, we could adapt the step width of the resistance change and the ON/OFF ratio. Additionally, we showed that the amount of resistance change during the SPM or RPM depends on the total time a voltage is applied irrespective of the number of pulses.

For the neural network simulations, the application of directly deposited Ta_2_O_5_ and WO_3_ layers does not lead to an increase in recognition accuracy or increased learning speed compared with the Al interlayer devices despite of the better linearity of the SPM and RPM curves of the WO_3_. A hyperparameter optimisation shows the influence of the pulse lenght and height on the SPM and RPM curves and the influence of their shape on the maximum accuracy. The ON/OFF ratio and the SPM steepness are identified as the most crucial for high accuracies. Furthermore, it was shown that using shorter update pulses leads to a slower initial increase of the recognition accuracy but a more stable learning process, with less oscillations. Based on this, we propose a new approach to implement a variable learning rate for resistive switching ReRAM devices by changing the pulse length that might be interesting for perceptron networks in the future.

In conclusion, we demonstrated how two fundamentally different learning rules for neural networks, STDP in SNN and gradient descent learning in ANN, could be realized in the same memristive devices.

## Data Availability Statement

The raw data supporting the conclusions of this article will be made available by the authors, without undue reservation.

## Author Contributions

All authors listed have made a substantial, direct and intellectual contribution to the work, and approved it for publication.

## Conflict of Interest

The authors declare that the research was conducted in the absence of any commercial or financial relationships that could be construed as a potential conflict of interest.
