# Supplementary material for: Exploring Area-Dependent Pr0.7Ca0.3MnO3-Based Memristive Devices as Synapses in Spiking and Artificial Neural Networks
Source: Front Neurosci. 2021 Jul 2;15:661261. doi: 10.3389/fnins.2021.661261 (PMC8282906; doi:10.3389/fnins.2021.661261)
Supplement: Supplementary file 1 [file Data_Sheet_1.docx]

Supplementary Material

1. **Network Structure**
   1. Layer Structure

The investigated multilayer perceptron consists of four neuron layers. The first neuron layer serves as the input layer and consists of 784 neurons, because the numbers of pixels per sample in the MNIST data set is 784. The last layer has 10 neurons, one for each class. In this case they resemble the numbers 0 – 9. The second and third layer of neuron are hidden layers and consist of 250 and 125 neurons, respectively. A sketch of the network structure can be found in Figure S1(A). Each two subsequent layers of neuron are connected by a fully connected weight layer, meaning that each neuron in one layer has exactly one connecting weight to every neuron in the other layer.

- 1. Memristive Array as weight layer matrix

To use memristive devices for fully connected weight layers e.g. in perceptron networks, many groups propose array structures of memristive devices (Xia and Yang, 2019), with each device resembling one weight in the layer. Each row in the array can be attributed to one input neuron and each column to one output neuron. This structure is depicted in Figure S1(B). In this memristive array, the input terminals of all devices in one row are connected and each rows receives one element of the input vector as a voltage signal. Each resistance produces a current depending on the input voltage and its resistance, resembling the multiplication of an element of the input vector with a single matrix element. The output terminals of all resistors in a column are also connected. On this column, the current through each device is summed up, yielding the output value of the vector matrix multiplication as a current following Kirchhoff’s Law.

For a forward pass, a voltage $V_{i}$is applied to every input neuron according to the gray scale value of respective pixel of the input sample. Using Ohm’s Law and Kirchhoff’s Law, the currents at the first hidden neuron layer are

$I_{j}= \sum_{i} V_{i} g_{ij}$, (1)

with $g_{ij}$ being the conductance of the PCMO device connecting the *i*^th^ input and the *j*^th^ output neuron. This resembles the result of the vector-matrix multiplication of the input neurons signal and the weight matrix. As the applied voltages $V_{i}$ and the weight conductances $g_{ij}$ are positive, also the output currents are all positive. Therefore, the activation of the output neurons must be shifted towards positive currents, e.g. by a constant current that is subtracted from the result of the vector-matrix multiplication for every output neuron. This current is the same for all output neurons and not a learning parameter. The output signal of these neurons is determined by a Rectified Linear Unit (ReLU) activation function in the proposed network.

1. **Hyperparameter Dependencies**

As described in section 2.4, the material stack and choice of the SET and RESET pulse voltage in the pulse measurement experiments has a strong influence on the shape of the resulting resistance curves. This also reflects on the fit parameters for function (1) for the respective measurements.

One important parameter is the maximum ratio between the resistance of a device in a fully on and off state, the ON/OFF ratio. Concerning the fit parameters, this value is expressed by the ratio of the maximum of the RPM resistance fit and the minimum of the SPM resistance fit. As described in section 2, for the RPM curves, the resistance is fitted by function (1) and for the SPM, the conductance is fitted. Therefore, the ON/OFF ratio is expressed as

$$\frac{ON}{OFF}ratio=\frac{R_{dep, max}}{R_{pot, min}}=R_{dep, max}*G_{pot, max}=ý_{dep}*ý_{pot}$$

For all investigated combinations of SET and RESET voltages, this value is plotted in Figure S2(A) for samples with an Al interlayer, Figure S2(B) for Ta_2_O_5_, and Figure S2(C). In general, the ON/OFF ratio that can be reached with the Ta_2_O_5_ interlayer is significantly lower than for the two other materials. Moreover, the SET and RESET pulse voltage have no strict influence on the ON/OFF ratio for all 3 types of devices. However, for the Al and especially for the WO_3_ interlayer sample, a trend to an increasing ON/OFF ratio with both increasing SET and RESET pulse voltage can be observed, except for the -2.6V potentiation curve for the WO_3_ samples, which yields a lower minimum resistance than the curves for lower voltages. For the Ta_2_O_5_ samples, an increasing ON/OFF ratio can be observed with an increase of RESET pulse voltage, except for the curve with a RESET pulse voltage of 1.6V.

For the fit parameter $\alpha_{SPM}$ in the fit of the SPM data and $\alpha_{RPM}$ the dependency on the SET pulse voltage and RESET pulse voltage, respectively, is investigated. For $\alpha_{SPM}$, which resembles the steepness of the initial increase of the conductance during the SPM, no clear voltage dependency can be found, as visible in Figure S2(B). Instead, the parameter rather depends on the interlayer material. For WO_3_ interlayer samples, the lowest values around 0.1 can be found, while for an Al interlayer the steepness is the highest with $\alpha_{SPM}$ values of around 0.3 to 0.35. The values for Ta_2_O_5_ are in between these two ranges.

In contrast, the $\alpha_{RPM}$ shows a trend to higher values with an increasing voltage in the RPM, as displayed in Figure S3(C). Yet, this trend is differently pronounced among the material stacks. For the WO_3_ interlayer samples $\alpha_{RPM}$ almost remains the same, while with a Ta_2_O_5_ interlayer, the strongest increase can be observed. The values for $\alpha_{RPM}$ however cannot be separated only by the different interlayer materials.

1. **Benchmarking against floating point weight based MLP**

Memristive devices are used in this work as a hardware replacement for floating point weights in a MLP in software. Therefore, the network performance using memristive devices is compared against the same network structure and learning rule using floating point weights. In contrast to the memristive weights, floating point weights have an infinite “ON/OFF ratio” as they can attain the value zero. They follow a linear update curve. With the sign update rule used here this means that the weights with the largest positive and negative weight are updated by the learning rate value in the positive or negative direction, respectively. The learning rate chosen for the floating point simulations is $1*{10}^{-4}$. Figure S3 shows the learning curve with floating point weights in comparison to the best performing learning curves of the three investigated material stacks. One can see that the maximum recognition accuracy reached with floating point weights is with about 96.3% higher than that of all the networks with memristive device that are investigated here. The maximum accuracy is reached much faster with the floating point weights. However, the speed of convergence with these weights strongly depends on the employed learning rate. With a lower learning rate the number of epochs to convergence with floating point weights can even be higher than for the memristive devices (not shown here).
The maximum accuracy of 96.3% with floating point devices is comparable to other software MLPs. (Baldominos et al., 2019) This shows in general the validity of the employed network structure and learning rule. The differences in the maximum recognition accuracies that are reached with memristive devices therefore stem from the specific behavior of these devices during the weight update, as discussed in the main paper.


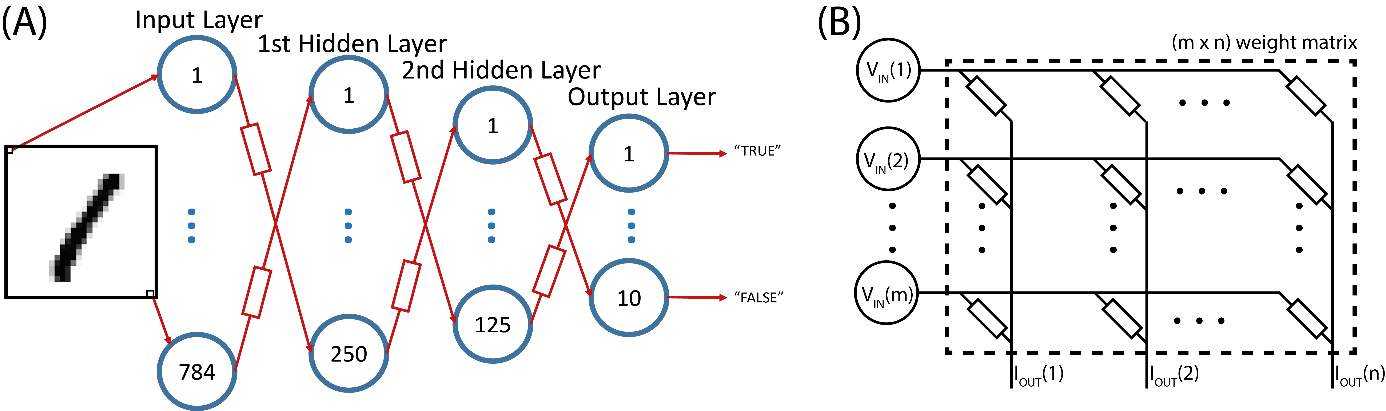


**Supplementary Figure S1.**

(A) Sketch of the multilayer perceptron network: It consists of four fully interconnected neuron layers with the displayed sizes. The first layer acts as the input layer and every neuron in this layer receives one of a sample from the MNIST data set as an input voltage according to its grayscale value. (B) Scheme of the matrix that has been used for the network simulations: Each layer of synapses consists of a matrix of PCMO memristive devices. With the input voltages applied to the rows of this matrix, the resulting currents through each device sum up on a column and yield the result of the vector matrix multiplication of the input voltage vector and the weight matrix.


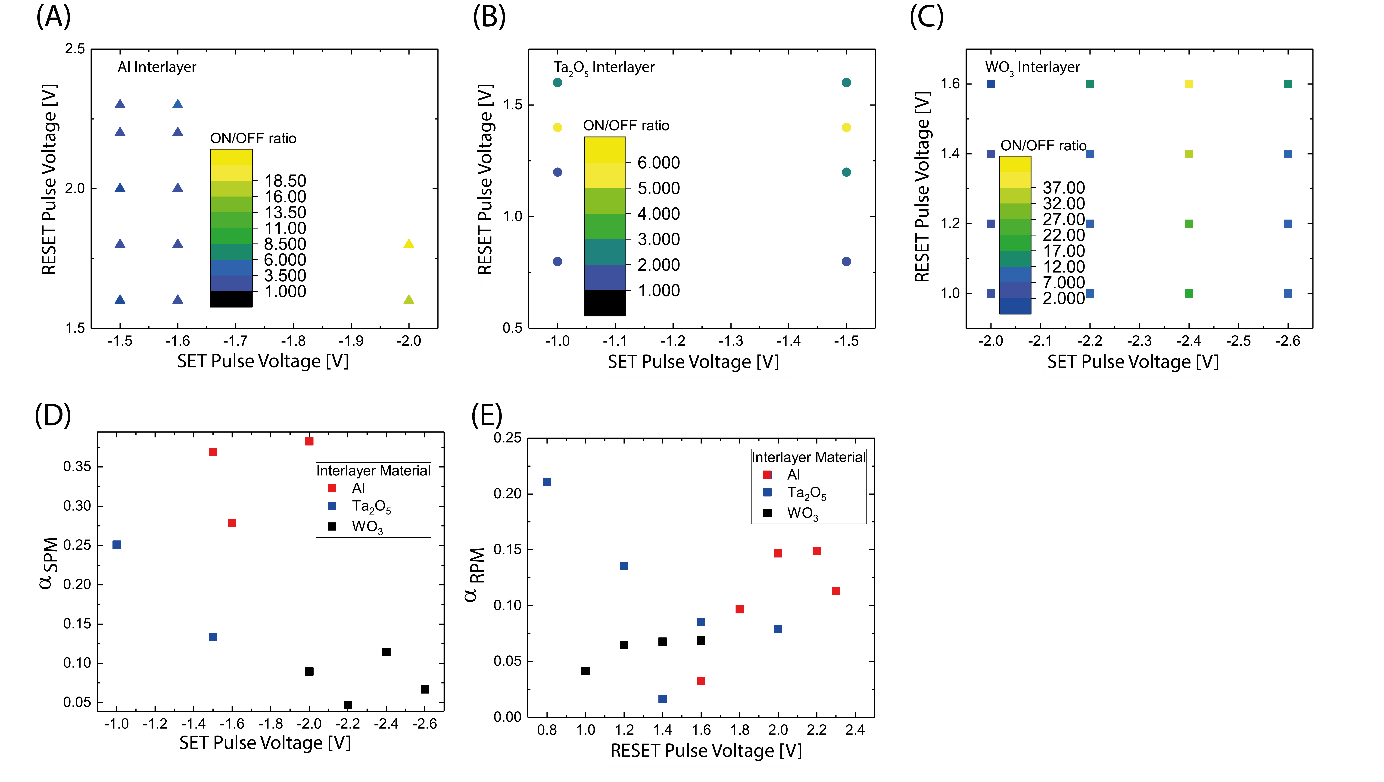
**Supplementary Figure S2.** (A)-(C): Dependence of ON/OF ratio (color-coded) on the SET and RESET pulse voltages. (A) Samples with an Al interlayer show the highest ON/OFF ratios for a SET pulse voltage of -2V, which again increases with the RESET pulse voltage. (B) For samples with a Ta_2_O_5_ interlayer, no clear dependency of the ON/OFF on the applied pulse voltages can be observed. (C) Samples with a WO_3_ interlayer show the highest ON/OFF ratios compared to the other materials and except for a SET pulse voltage of -2.6V a clear trend of an increasing ON/OFF ratio with SET and RESET pulse voltages. (D) Dependence of fit parameter $\alpha_{SPM}$ on the SET pulse voltage. $\alpha_{SPM}$ mostly depends on the interlayer material with Al yielding the highest, Ta_2_O_5_ intermediate and WO_3_ the lowest values. (E) Dependence of fit parameter $\alpha_{RPM}$ on the RESET pulse voltage. $\alpha_{RPM}$ increases with the RESET pulse voltage. The steepness of the increase depends on the interlayer material.


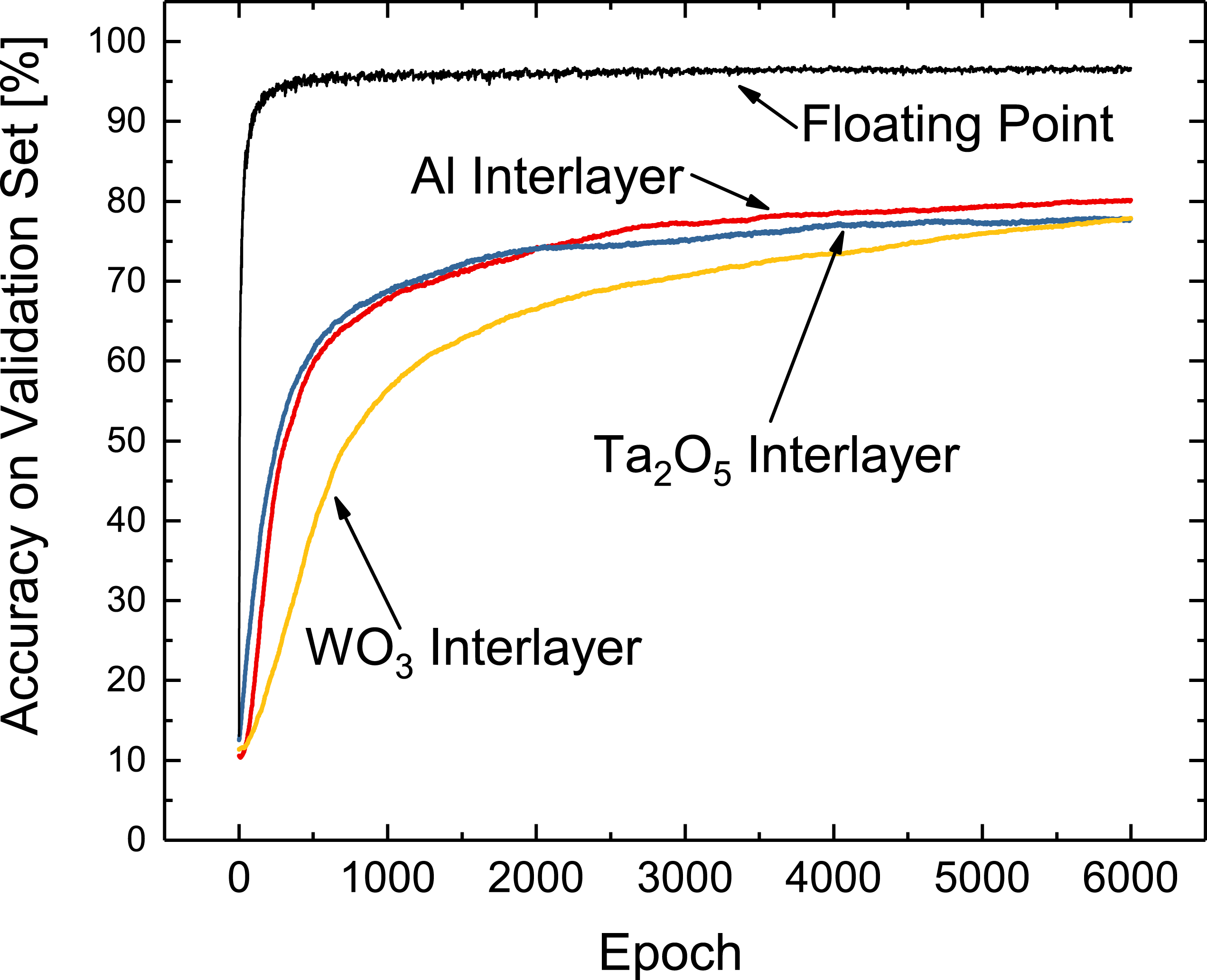


Supplementary Figure S3. Comparison of the best performing learning curves with memristive devices with a benchmark with floating point weights. The benchmark test yields a maximum recognition accuracy of about 96.3% while the networks with PCMO ReRAM devices reach a 12% (Al interlayer) to 20% (Ta_2_O_5_ and WO_3_ interlayer) lower maximum accuracy.

References:

Baldominos, A., Saez, Y., and Isasi, P. (2019). A survey of handwritten character recognition with MNIST and EMNIST. *Appl. Sci.* 9. doi:10.3390/app9153169.

Xia, Q., and Yang, J. J. (2019). Mewmristive crossbar arrays for brain-inspired computing. *Nat. Mater.* 18. doi:10.1038/s41563-019-0291-x.
